# Xinfeng Capsule inhibits oxidative stress via regulating the PPARγ/ Hmgcs2 signaling pathway in lung tissue of adjuvant arthritis rats

**DOI:** 10.3389/fphar.2025.1599745

**Published:** 2025-10-08

**Authors:** Ping-Heng Zhang, En-Sheng Chen, Jian Liu, Chang-Hong Xiao

**Affiliations:** 1 Department of Nephrology and Rheumatology, The Affiliated TCM Hospital of Guangzhou Medical University, Guangzhou, China; 2 Rheumatology and Immunology Department, Southern Medical University Hospital of Integrated Traditional Chinese and Western Medicine, Southern Medical University, Guangzhou, China; 3 Department of Rheumatology and Immunology, First Affiliated Hospital of Anhui University of Traditional Chinese Medicine, Hefei, China

**Keywords:** Xinfeng Capsule, adjuvant arthritis, lung injury, oxidative stress, PPARγ, HMGCS2

## Abstract

**Background:**

Xinfeng Capsule (XFC) is a traditional Chinese medicine compound preparation that has been clinically used to treat rheumatoid arthritis (RA) for more than 20 years. It has demonstrated clear therapeutic effects, including improving pulmonary function and reducing lung injury in patients with RA. However, the precise mechanism underlying its protective effect against lung injury remains unclear. This study aims to explore the potential mechanisms of XFC in the treatment of lung injury.

**Methods:**

Liquid chromatography-mass spectrometry (LC-MS) analysis was conducted to determine the chemical composition of XFC. Proteomic and bioinformatic analyses of differentially expressed proteins (DEPs) in rat lung tissue were performed using tandem mass tag labeling. A rat adjuvant arthritis (AA) model was established using Freund’s complete adjuvant to observe pathological changes in synovial and lung tissues, as well as alterations in lung function. In addition, a cell model was constructed by inducing lung fibroblasts with transforming growth factor-β1 (TGF-β1) to investigate the effects of XFC-containing serum on oxidative stress and pulmonary fibrosis through the peroxisome proliferator-activated receptor gamma (PPARγ)/3-hydroxy-3-methylglutaryl-CoA synthase 2 (HMGCS2) pathway.

**Results:**

LC-MS analysis identified a total of 867 compounds in XFC, of which 25 unique compounds were closely associated with pulmonary fibrosis and lung injury. Proteomic analysis suggested that XFC may regulate PPAR signaling pathway-related proteins and alleviate lung injury in AA rats. Animal experiments showed that XFC significantly inhibited immune inflammation, synovial hyperplasia, and oxidative stress in AA rats, while reducing lung injury and improving lung function. Furthermore, XFC-containing serum suppressed TGF-β1–induced proliferation of lung fibroblasts, promoted PPARγ expression, and significantly decreased the levels of interleukin-6, tumor necrosis factor-α, reactive oxygen species, nicotinamide adenine dinucleotide phosphate oxidase 4, HMGCS2, collagen type I α 1, collagen type III α 1, and α-smooth muscle actin (*P* < 0.01). In addition, XFC partially reversed the effects of the PPARγ antagonist GW9662, activated the PPARγ signaling pathway, inhibited oxidative stress and inflammatory responses, and exerted anti-fibrotic effects similar to those of the PPARγ agonist rosiglitazone.

**Conclusion:**

XFC inhibits inflammation and oxidative stress by regulating the PPARγ/HMGCS2 pathway, thereby attenuating fibrosis and alleviating lung injury.

## 1 Introduction

Rheumatoid arthritis (RA) is a complex autoimmune disease with multifaceted etiology and mechanisms. The global prevalence of RA is estimated to be between 0.5% and 1% (Finckh et al., 2022). As the disease progresses and recurs, many patients develop pathological changes in tissues and organs beyond the joints. Notably, the lungs are frequently affected in this extra-articular manifestation, involving the lung parenchyma, pleura, and blood vessels. This leads to diverse pathological alterations, including interstitial lung lesions, rheumatoid nodules, and pleuritis (Akiyama and Kaneko, 2022). Among these complications, interstitial lung disease (ILD) is the most common form of pulmonary involvement in RA patients (Samhouri et al., 2022). The reported prevalence of ILD in RA patients varies widely across different cohorts, largely due to differences in detection frequency and diagnostic criteria. Earlier studies estimated that secondary ILD occurs in 1.8%–67% of RA patients (Dai et al., 2021; Huang et al., 2020). This condition not only adversely affects the quality of life of the patients but also imposes a considerable social and economic burden. Unfortunately, the early stages of RA-associated lung injury often lack obvious clinical symptoms and are instead characterized by a gradual decline in lung function. This insidious onset frequently results in underrecognition and delayed intervention. At present, the precise pathogenesis of lung injury secondary to RA remains poorly understood, and therapeutic options are limited. This knowledge gap highlights the urgent need for effective treatments for this debilitating complication.

The peroxisome proliferator-activated receptor γ (PPARγ) signaling pathway has emerged as a key regulator of inflammation, oxidative stress, and fibrosis—central pathological processes in lung injury (Liu et al., 2020). Activation of PPARγ exerts both anti-inflammatory and anti-fibrotic effects in various lung disease models (Li et al., 2024). Notably, 3-hydroxy-3-methylglutaryl-CoA synthase 2 (HMGCS2), a critical enzyme in ketogenesis, has recently been identified as a downstream target regulated by PPARγ signaling. Dysregulation of the PPARγ/HMGCS2 axis has been implicated in promoting oxidative stress and cellular damage in extrapulmonary tissues (Hazra et al., 2008). However, its specific role and therapeutic potential in RA-associated lung injury remain largely unexplored, representing both a major knowledge gap and a promising therapeutic target.

Traditional Chinese medicine (TCM) has gained significant attention due to its complex bioactive components and diverse therapeutic targets. Among TCM formulations, Xinfeng Capsule (XFC) is a noteworthy compound preparation produced under strict quality control standards (Wang et al., 2014). XFC has been registered in China (Anhui Medicine System Z20050062) and granted a national invention patent (ZL201310011369.8). Comprising Astragali Radix (*Astragalus membranaceus* (Fisch.) Bge. var. *mongholicus* (Bge.) Hsiao or *A. membranaceus* (Fisch.) Bge.), Coicis Semen (*Coix lacryma-jobi* L. var. *ma-yuen* (Roman.) Stapf), Tripterygium wilfordii Hook. f., and Scolopendra (*Scolopendra subspinipes mutilans* L. Koch) at a ratio of 20:20: 10:1, XFC has shown considerable therapeutic promise(Gao et al., 2020). In a previous large-scale, multicenter, randomized, double-blind clinical trial conducted by our team (Jian et al., 2015), XFC demonstrated significant efficacy in alleviating joint symptoms and improving lung function in RA patients (Sun et al., 2016; Wan et al., 2016; Yali et al., 2015). Furthermore, animal studies showed that XFC improved lung injury in adjuvant arthritis (AA) rats, while cell-based experiments revealed its ability to inhibit lung fibroblast proliferation (Wan et al., 2017). Despite these encouraging findings, the underlying molecular mechanisms—particularly the potential involvement of the PPARγ/HMGCS2 pathway—remain to be clarified.

With advances in molecular biology, proteomic analysis has become a powerful tool in biomedical research, offering a broad perspective for exploring the mechanisms of TCM. In this study, we employed an unbiased, tandem mass tag (TMT)-based proteomic approach to identify novel targets of XFC in the lungs of AA rats. We hypothesized that XFC alleviates RA-associated lung injury by modulating the PPARγ/HMGCS2 signaling pathway to reduce oxidative stress. This study was therefore designed to comprehensively characterize the chemical composition of XFC, validate its efficacy in improving lung function and pathology in AA rats, identify differentially expressed proteins (DEPs) in lung tissue, and mechanistically confirm both *in vitro* and *in vivo* that its protective effects are mediated through the PPARγ/HMGCS2 signaling axis. These findings provide novel HMGCS2 insights and additional experimental evidence for a deeper understanding of the pharmacological mechanisms of XFC.

## 2 Materials and methods

### 2.1 Preparation and analysis of Xinfeng Capsule components using liquid chromatography-mass spectrometry (LC-MS)

XFC is a compound formulation rooted in traditional Chinese medicine. The raw materials used in this preparation were procured from Lejialaopu Pharmacy (Hefei, Anhui Province) and authenticated by Professor Li from the First Affiliated Hospital of Anhui University of Chinese Medicine. All herbal components comply with the specifications outlined in the 2020 edition of the Chinese Pharmacopoeia and were manufactured in strict accordance with national regulatory standards. The formulation consists of *A. membranaceus* (Batch No. 2208015, Inner Mongolia, China), *C. lacryma-jobi* L. var (Batch No. YPC2F0012, Fujian, China), *S. subspinipes mutilans* L. Koch (Batch No. 2207008, Hubei, China), and *Tripterygium wilfordii Hook. f* (Batch No. YPT1G0005, Guizhou, China), with a defined dosage ratio of 20:20:10:1. In prior research, our team established a high-performance liquid chromatography (HPLC)-based fingerprint profile, which demonstrated that XFC possesses a well-controlled manufacturing process and rigorous quality control standards (Gao et al., 2020).

Preparation of XFC extract: Accurately weigh 4.0 g of XFC powder and transfer it to a stoppered conical flask (100 mL) containing 40 mL of methanol. Perform ultrasonic extraction at room temperature for 40 min, followed by filtration through filter paper. Concentrate the filtrate to dryness under reduced pressure, then reconstitute in 2 mL of methanol. Centrifuge the resulting solution at 12,000 rpm for 10 min at 4 °C, collect the supernatant, and filter it through a 0.22 μm nylon membrane filter. All prepared solutions should be stored at 4 °C and protected from light.

LC-MS Analysis: LC separation was performed using a Thermo Vanquish ultra-high performance liquid chromatography system (Thermo Fisher Scientific, United States) equipped with an ACQUITY UPLC^®^ HSS T3 column (2.1 × 150 mm, 1.8 µm) (Waters, Milford, MA, United States). The mobile phase consisted of acetonitrile and water containing 0.1% formic acid, and gradient elution was carried out at a flow rate of 0.25 mL/min. The column temperature was maintained at 40 °C, and the injection volume was 2 μL. Mass spectrometry analysis was conducted using an electrospray ionization (ESI) source in both positive and negative ion modes. ESI source parameters were as follows: sheath gas flow rate, 30 arb. units; auxiliary gas flow rate, 10 arb. units; positive ion spray voltage, 3.50 kV; negative ion spray voltage, −2.50 kV; capillary temperature, 325 °C. Full-scan MS was performed in the first stage at a resolution of 60,000 over a mass range of m/z 100–1,000. For tandem mass analysis, HCD fragmentation was employed with a collision energy of 30%, secondary resolution of 15,000, and the top four precursor ions selected for fragmentation prior to signal acquisition. Dynamic exclusion was applied to eliminate redundant MS/MS spectra. The detailed chromatographic analysis conditions are provided in the supplementary materials.

### 2.2 Rationale for XFC dose selection

The high and low doses of XFC used in this study (648 mg/kg/day and 162 mg/kg/day, respectively) were determined according to the body surface area conversion principle commonly applied in pharmacological studies. Based on this method, the conversion factor between rats and humans is 6.3, yielding a rat-equivalent daily dose of 324 mg/kg/day. The high and low doses were set at twice and half this value, respectively.

### 2.3 Animal experiments and treatment

After 1 week of adaptive feeding, 36 Sprague–Dawley (SD) rats were included in the study and randomly assigned to the following groups: Control, AA model, XFC treatment (high- and low-dose, H-XFC and L-XFC), and Leflunomide (LEF) positive control group. Each group consisted of six rats. The AA model was established as described previously (Wan et al., 2013), after which treatment commenced. XFC was ground into a fine powder and formulated into 0.068 g/mL (H-XFC) and 0.017 g/mL (L-XFC) suspensions for intragastric administration in the XFC group. LEF was similarly prepared into a 0.095 mg/mL suspension. The AA group received intragastric administration of 0.9% normal saline. All groups received a daily intragastric dose of 1 mL/100 g body weight at 2 p.m. for 4 weeks. This study was approved by the Animal Ethics Committee of Anhui University of Traditional Chinese Medicine (Approval No. AHUCM-rats-2021081).

The joint swelling of the rat (paw volume) and arthritis scores were recorded every 5 days. Twenty-four hours after the final administration, rats were anesthetized via intraperitoneal injection of pentobarbital, and the lung function was assessed using an animal lung function apparatus. The trachea was rapidly isolated, and bronchoalveolar lavage was performed with 10 mL/kg of normal saline, repeated three times. The lavage fluid was collected and centrifuged at 1,500 r/min for 10 min at 4 °C, and the supernatant was transferred into Eppendorf (EP) tubes and stored at −80 °C. Finally, rats were euthanized, and lung and joint synovial tissues were collected.

### 2.4 Hematoxylin–eosin (H&E) and Masson staining

The left ankle joint synovial tissue and lung tissue were embedded in paraffin and sectioned at 5 μm thickness. Sections of both tissues were stained with H&E, while lung tissue sections were also stained with Masson’s staining. Pathological changes of joint synovial and lung tissues were observed under an optical microscope. The extent of alveolar inflammation was evaluated using the Szapiel method, consistent with previous studies (Zhang P. H. et al., 2023). The fibrotic area was quantified using ImageJ image analysis software.

### 2.5 Proteomic analysis

Proteins were extracted from rat lung tissue, and concentrations were determined using the bicinchoninic (BCA) kit. For FASP enzymatic hydrolysis, 200 μg of protein was used. The resulting digested samples were freeze-dried and stored at −80 °C.

For labeling, 100 μg of peptides from each sample were equilibrated in 50 μL of 100 mM triethyl ammonium bicarbonate buffer at room temperature. The TMTpro reagent was equilibrated to room temperature, dissolved in anhydrous acetonitrile, incubated for 5 min, and centrifuged. Subsequently, 10 μL of TMTpro reagent was added to each sample in 1.5 mL EP tubes for labeling. Labeling was performed for 1 h, and the reaction was terminated with 5% hydroxylamine for 15 min. The labeled samples were subjected to chromatographic separation using the high-performance liquid chromatography (Agilent Zorbax Extend-C18 narrow diameter)column and then loaded onto the EASY-nLC 1,200 liquid-phase system (Thermo Fisher) at a flow rate of 300 nL/min for separation. The peptides were further analyzed using a Q Exactive HF mass spectrometer (Thermo Fisher).

Spectral data were analyzed with Proteome Discoverer software (Version 2.4, Thermo Fisher Scientific, USA). DEPs were identified using thresholds of q < 0.05 and |log_2_(Fold change [FC])| >1. The Gene Ontology (GO) database was applied to classify DEPs into biological processes (BP), cellular components (CC), and molecular functions (MF). Kyoto Encyclopedia of Genes and Genomics (KEGG) pathway analysis was used to identify enriched pathways. Protein–protein interaction (PPI) networks of DEPs were constructed using the STRING database.

### 2.6 Preparation of XFC-containing serum

Ten male SD rats were randomly divided into a normal serum group and a drug-containing serum group, with five rats in each group. The normal serum group received 0.9% physiological saline by gavage, while the drug-containing serum group received an XFC suspension (2.4 g/100 g) by gavage. The detailed methodology can be found in our previously published protocols (Sun et al., 2023).

### 2.7 Cell culture and cell counting kit-8 (CCK-8) assay

HFL-1 lung fibroblasts (purchased from the cell bank of the Chinese Academy of Sciences, Shanghai, China) were cultured in F12-K medium supplemented with 10% fetal bovine serum and 1% penicillin–streptomycin. The cells were maintained in an incubator at 37 °C, 5% carbon dioxide, and saturated humidity. A cell model was established by treating the cells with 50 ng/mL transforming growth factor-β1 (TGF-β1). Subsequently, the cells were exposed to different concentrations of XFC-containing serum (10%, 20%, and 40%) for 24, 48, and 72 h, respectively. Cell viability was assessed using the CCK-8 assay kit (Sigma, USA) according to the manufacturer’s instructions, and the optimal intervention concentration of XFC-containing serum was determined for subsequent experiments. To further investigate the role of activated PPARγ in TGF-β1-induced oxidative stress in HFL-1 fibroblasts, cells were treated with rosiglitazone (RSG) (10 μmol/L) or GW9662 (a PPARγ antagonist, 5 μmol/L).

### 2.8 Enzyme-linked immunosorbent assay (ELISA)

Bronchoalveolar lavage fluid and the supernatant from HFL-1 cells were used to quantify the levels of IL-1β, TNF-α, and IL-6. The enzyme-linked immunosorbent assay (ELISA) kits demonstrated high sensitivity, with detection limits below 1.0 pg/mL for all cytokines. A standard curve was generated by plotting the standard sample concentrations (x-axis) against their corresponding optical density (OD) values (y-axis). The correlation coefficient between the measured and expected concentrations was ≥0.9900, indicating high assay accuracy and reliability. Cytokine concentrations in the samples were calculated using the regression equation from the standard curve. All ELISA reagents were obtained from Solbo Biotechnology Co., Ltd. (Beijing, China).

### 2.9 Flow cytometry (FCM) assay

HFL-1 cells were seeded into six-well plates at a density of 2 × 10^4^ cells/mL, with 3 mL per well, and cultured for 48 h. The cells were then harvested by trypsin digestion, and the supernatant was removed by centrifugation. The cell pellet was resuspended in 0.5 mL of serum-free culture medium in an EP tube, followed by the addition of 0.5 µL of 2′,7′-dichlorofluorescein diacetate. After incubation at 37 °C for 20 min, the cells were centrifuged, the supernatant was discarded, and the cells were washed three times with serum-free medium before being resuspended for analysis. For FCM, scatter plots were generated with forward scatter on the x-axis and side scatter on the y-axis. By adjusting FSC and SSC thresholds, target cells were identified and gated. The gated population was analyzed by switching the x-axis to FL1 and generating histograms of fluorescence intensity. N-acetylcysteine served as the negative control, while tert-butyl hydroperoxide served as the positive control. The negative control was used to determine background fluorescence, and the positive control validated assay sensitivity and reliability. Within the flow cytometer’s analysis software, the mean fluorescence intensity (MFI) of the gated population was calculated. MFI reflects intracellular reactive oxygen species (ROS) levels, with higher MFI indicating increased ROS.

### 2.10 Western blotting

Lung tissues were homogenized in radioimmunoprecipitation assay buffer, and protein concentrations were measured using a BCA protein assay kit. Sodium dodecyl sulfate-polyacrylamide gel electrophoresis was used to separate equal amounts of protein, which were then transferred to membranes and blocked. After washing, membranes were incubated overnight at 4 °C with primary antibodies against Fabp5 (1:1,000), Pltp (1:1,000), Me1 (1:1,000), and β-actin (1:5,000). After three washes with tris-buffered saline (TBST) (5 min per wash), the membranes were incubated with horseradish peroxidase-conjugated secondary antibody (1:10,000) for 1 h at room temperature. Following three additional washes with TBST (5 min each), protein bands were visualized using ECL Plus luminol reagent, and images were captured by scanning. β-actin was used as the internal loading control. Relative protein expression was quantified by normalizing target protein band intensities to β-actin using ImageJ software.

### 2.11 Quantitative real-time polymerase chain reaction (qRT-PCR)

Total RNA was extracted from rat lung tissue using the TRIzol kit. Total RNA concentration and purity were assessed spectrophotometrically. Complementary DNA was synthesized from total RNA using reverse transcriptase, according to the manufacturer’s protocol. qRT-PCR quantified the mRNA expression levels of collagen type I alpha 1 (*COL1A1*) and collagen type III alpha 1 (*COL3A1*). Primer sequences are listed in Supplementary Table S1. Relative fold changes in gene transcription were determined using the 2^−ΔΔCt^ method.

### 2.12 Immunofluorescence (IF)

Paraffin-embedded lung sections were dewaxed and rehydrated. Antigen retrieval was performed using trypsin at 37 °C for 15 min, followed by three washes. Sections were blocked with 4% bovine serum albumin at room temperature for 60 min. Primary antibodies against PPARα, PPARγ, alpha-smooth muscle actin (α-SMA), HMGCS2, and NADPH oxidase 4 (NOX4) (1:100 dilution) were applied overnight at 4 °C. After washing three times with phosphate bovine serum (PBS), sections were incubated with fluorescent secondary antibody (1:200 dilution) for 1 h in the dark. Following another PBS wash, sections were mounted with antifluorescence medium and immediately scanned using a confocal laser fluorescence microscope.

### 2.13 Immunohistochemistry

Rat lung tissue sections were dewaxed, rehydrated, and subjected to high-pressure repair using citric acid buffer (pH 6.0). After PBS washing, endogenous peroxidase activity was blocked with 30 mL/L hydrogen peroxide. Primary antibodies against α-SMA, HMGCS2, NOX4, and superoxide dismutase (SOD) (1:100 dilution) were applied overnight at 4 °C.

The following day, sections were reheated, washed with PBS, and incubated with secondary antibody (1:200 dilution) at 37 °C for 30 min. Visualization was achieved using DAB staining, followed by hematoxylin counterstaining, differentiation, washing, and sealing. Positive signals were observed under a light microscope. The integrated OD values of α-SMA, HMGCS2, NOX4, and SOD were quantified using ImageJ software.

### 2.14 Statistical analysis

All statistical analyses were performed using the Statistical Package for Social Sciences 22.0 software. Prior to one-way analysis of variance (ANOVA), the assumptions of normality and homogeneity of variance were verified. Normality was tested using the Shapiro–Wilk test, and homogeneity of variance was evaluated using Levene’s test. For data that met these assumptions, ANOVA was performed, followed by Tukey’s *post hoc* test. If homogeneity of variance was violated, Welch’s ANOVA followed by the Games–Howell test was applied. Results were expressed as mean ± standard deviation. P < 0.05 was considered statistically significant. Graphs were generated using GraphPad Prism 8.0 software.

## 3 Results

### 3.1 Identification of XFC components through LC-MS analysis

**FIGURE 1:**
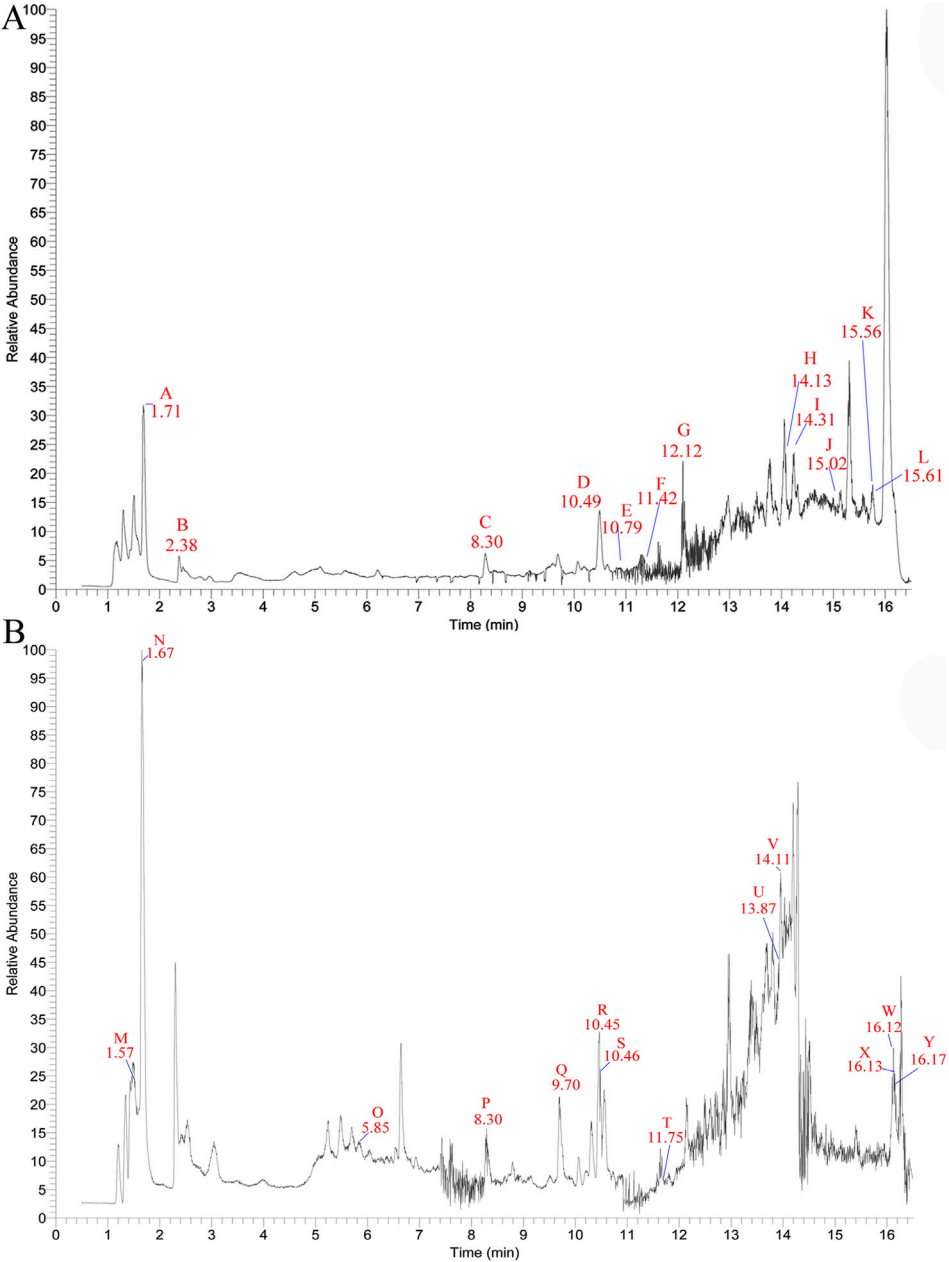
The total ion chromatogram (TIC) of XFC in negative ion mode **(A)** and positive ion mode **(B)** by LC-MS analysis.

To comprehensively analyze the chemical composition and potential pharmacological effects of XFC, we performed LC-MS on XFC extracts. Based on reference data from the mzCloud database (https://www.mzcloud.org/Stats), the Human Metabolome Database (http://www.hmdb.ca), and LipidMaps (http://www.lipidmaps.org), together with the results of precise mass-to-charge ratio (m/z) measurements and secondary fragment analysis, we identified a total of 867 compounds in XFC. In addition, 25 unique compounds were identified through secondary metabolite profiling and comparison with literature data, and these are speculated to be closely associated with pulmonary fibrosis and lung injury. Notable compounds include quercetin, coumarin, ononin, astragaloside IV, astragaloside A, soybean apogenol A, epicatechin, cervetol, and kaempferol (see Table 1 for details). The base peak chromatogram for LC-MS analysis of the XFC extract is shown in Figure 1.

**TABLE 1:** LC-MS analysis of XFC.

Peak name	Name	MZ	RT(min)	Formula	Precursor-type
A	Quercetin 3-arabinoside	433.08	1.71	C20H18O11	[M-H]-
B	Gancaonin A	352.13	2.38	C21H20O5	[M]-
C	Caffeic acid	179.03	8.30	C9H8O4	[M-H]-
D	Ferulate	193.05	10.49	C10H10O4	[M-H]-
E	Astragaloside IV	783.46	10.79	C41H68O14	[M-H]-
F	Kaempferol	285.04	11.42	C15H10O6	[M-H]-
G	Formononetin	266.96	12.12	C16H12O4	[M-H]-
H	Palmitic acid	255.07	14.14	C16H32O2	[M-H]-
I	Quercetin 3,4′-diglucoside	625.51	14.31	C27H30O17	[M-H]-
J	Quercetin	301.18	15.02	C15H10O7	[M-H]-
K	Kaempferol 3,7-diglucoside	609.51	15.56	C27H30O16	[M-H]-
L	Stearic acid	283.26	15.61	C18H36O2	[M-H]-
M	Betaine	118.12	1.57	C5H11NO2	[M + H]+
N	coumarin-SAHA	347.16	1.67	C18H22N2O5	[M + H]+
O	Kaempferol 3-O-β-D-xyloside	419.09	5.85	C20H18O10	[M + H]+
P	Epicatechin	291.08	8.31	C15H14O6	[M + H]+
Q	Ononin	431.12	9.70	C22H22O9	[M + H]+
R	Luteolin	287.05	10.45	C15H10O6	[M + H]+
S	Berberine	336.12	10.46	C20H18NO4	[M + H]+
T	Astragaloside A	785.98	11.75	C41H68O14	[M + H]+
U	Soyasapogenol A	474.37	13.87	C30H50O4	[M]+
V	Celastrol	451.28	14.11	C29H38O4	[M + H]+
W	Soyasapogenol B	441.37	16.12	C30H50O3	[M + H-H2O]+
X	Campesterol	383.36	16.13	C28H48O	[M + H-H2O]+
Y	Lupeol	427.39	16.17	C30H50O	[M + H]+

### 3.2 XFC alleviates paw swelling and arthritis scores in AA rats

**FIGURE 2:**
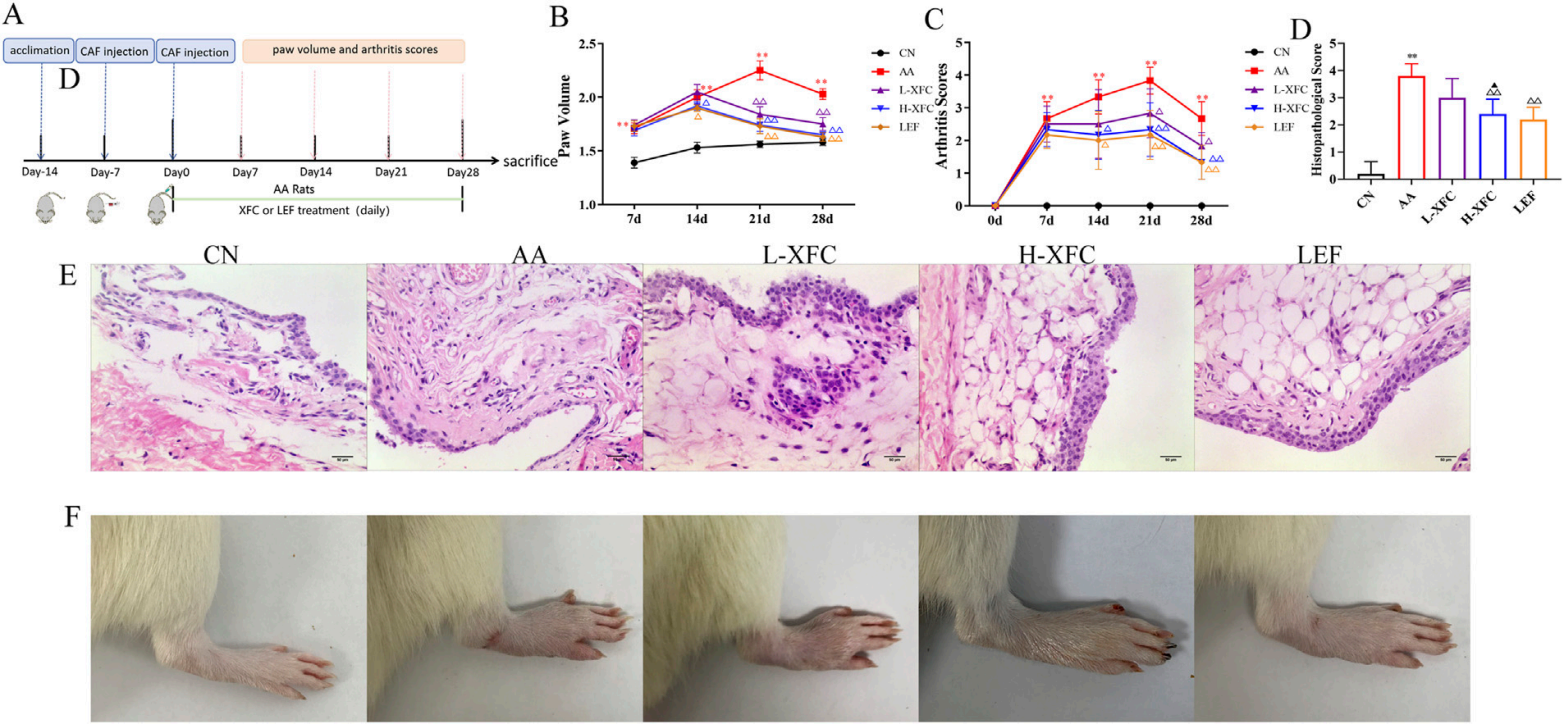
XFC reduced paw volume and arthritis scores of adjuvant arthritis rats. **(A)** Schematic image for the establishment of AA mouse model with treatment. **(B,C)** Paw volume and arthritis scores of AA rats (n = 6) treated with different doses of (L, H)-XFC (162 mg/kg/d, 648 mg/kg/d). **(D)** The analysis of joint synovium histopathological score. **(E)** H&E staining of joint synovium in rats (n = 3). **(F)** Morphological changes of the ankle joint in rats (n = 3). **P < 0.01 vs. CN group, ^△^ *P* < 0.05, ^△△^ *P* < 0.01 vs. AA group. ^▲^ *P* < 0.05 vs. L-XFC group. CN group: SD rats. AA group: Rats successfully modeled by the Fauquier complete adjuvant method. L-XFCgroup: Rats with AA treated by low-dose XFC intragastric administration. H-XFCgroup: Rats with AA treated by high-dose XFC intragastric administration. LEF: Rats with AA treated by leflunomide.

By the second day after Freund’s complete adjuvant (FCA) injection, rats exhibited visible swelling of the toes. By the seventh day, the adjuvant-induced arthritis (AA) model was successfully established. A booster immunization was then administered via injection of 0.05 mL of CFA at the base of the tail. Paw volume and arthritis scores were recorded weekly. Compared with the control group, the AA group showed significantly greater paw volume and arthritis scores, which peaked on day 21 after induction (Figures 2A–C).

From day 14 post-FCA injection, AA rats received different doses of XFC (low- and high-doses) or LEF for therapeutic intervention. Compared with the AA group, both LEF and H-XFC significantly reduced paw swelling and arthritis scores beginning on day 14. L-XFC also showed significant improvements, though only from day 21. No statistically significant differences were observed between the LEF-treated and H-XFC-treated groups (Figures 2A–C). Histopathological analysis of joint synovial tissue in AA rats revealed marked synovial proliferation and extensive inflammatory cell infiltration. Treatment with either XFC or LEF reduced synovial hyperplasia and inflammatory infiltration. These findings indicate that H-XFC exerts stronger therapeutic efficacy than L-XFC; therefore, H-XFC was selected as the optimal dosage for subsequent experiments (Figures 2D–F).

### 3.3 XFC alleviates lung injury in AA rats

We next examined the effects of XFC on lung injury by assessing histopathology, pulmonary function, fibrosis, and inflammation in AA rats. Following model establishment, rats received XFC (low or high dose) or LEF for 15 consecutive days.

**FIGURE 3:**
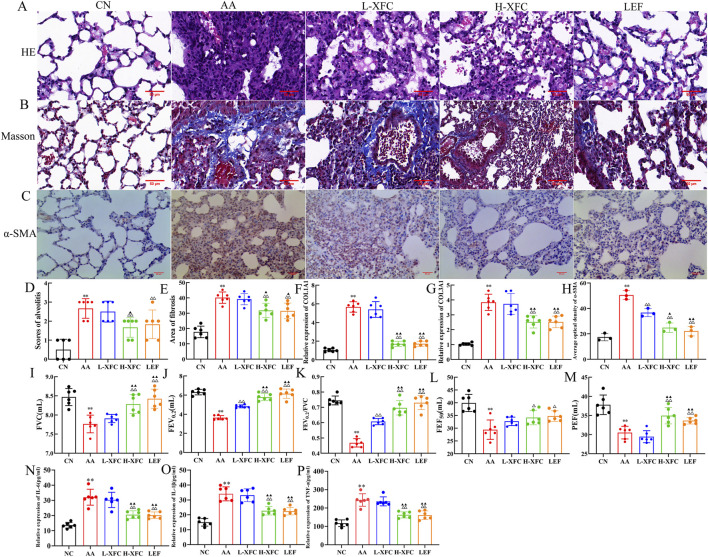
The effect of XFC on lung injury in AA rats. **(A)** H&E staining (×400). **(B)** Masson staining (×400). **(C)** Immunohistochemical representative image of Alpha-Smooth Muscle Actin(α-SMA) (n = 3). **(D)** The area of fibrosis in lung tissues(n = 3). **(E)** Scores of alveolitis of lung tissues(n = 3). **(F)** the relative expression of α-SMA(n = 3). **(G,H)** the relative expression of Collagen Type I Alpha-1 Chain(COL1A1) and Collagen type III Alpha-1 Chain(COL3A1)mRNA(n = 6). **(I–M)** XFC could improve the lung function in AA rats (FVC: forced vital capacity, FEV_0.2_: forced expiratory volume at 0.2 s, FEV_0.2_/FVC: forced expiratory volume at 0.2 s/forced vital capacity, PEF: peak expiratory flow, FEF_50_: expiratory flow 50%) (n = 6). **(N–P)** Analysis of cytokine (IL-1β, IL-6, and TNF-α) in bronchoalveolar lavage fluid by ELISA(n = 6). **P < 0.01 vs. CN group, ^△^ *P* < 0.05or^△△^ *P* < 0.01vs AA group, ^▲^ *P* < 0.05 or ^▲▲^ *P* < 0.01 vs. L-XFC group. CN group: SD rats. AA group: Rats successfully modeled by the Fauquier complete adjuvant method. L-XFCgroup: Rats with AA treated by low-dose XFC intragastric administration. H-XFCgroup: Rats with AA treated by high-dose XFC intragastric administration. LEF: Rats with AA treated by leflunomide.

Compared with the AA group, both H-XFC and LEF treatment markedly reduced inflammatory cell infiltration and collagen deposition in lung tissue. Improvements were also observed in the alveolar structure, characterized by a more regular architecture, enlarged alveolar spaces, and thinner septa. No significant differences were observed between the H-XFC and LEF groups. By contrast, L-XFC treatment did not significantly improve pathological changes (Figures 3A,B,D,E).

Relative to the AA group, H-XFC and LEF also significantly decreased the expression of fibrosis markers (α-SMA, COL1A1, and COL3A1) and reduced levels of inflammatory cytokines (IL-1β, IL-6, and TNF-α) in bronchoalveolar lavage fluid (Figures 3C,F–H,N–P). In addition, pulmonary function parameters—including FVC, FEV0.2, FEV0.2/FVC, PEF, and FEF50—were significantly improved in both H-XFC- and LEF-treated groups (Figures 3I–M). Again, no significant differences were noted between H-XFC and LEF, while L-XFC produced no marked improvements.

### 3.4 Proteomics analysis of DEPs in the lung tissue of AA rats

To investigate the potential protein targets of XFC in lung injury, we performed TMT-based proteomic analysis of lung tissues from AA rats. A total of 7,214 proteins were identified, of which 5,680 met the criteria for quantitative analysis.

**FIGURE 4:**
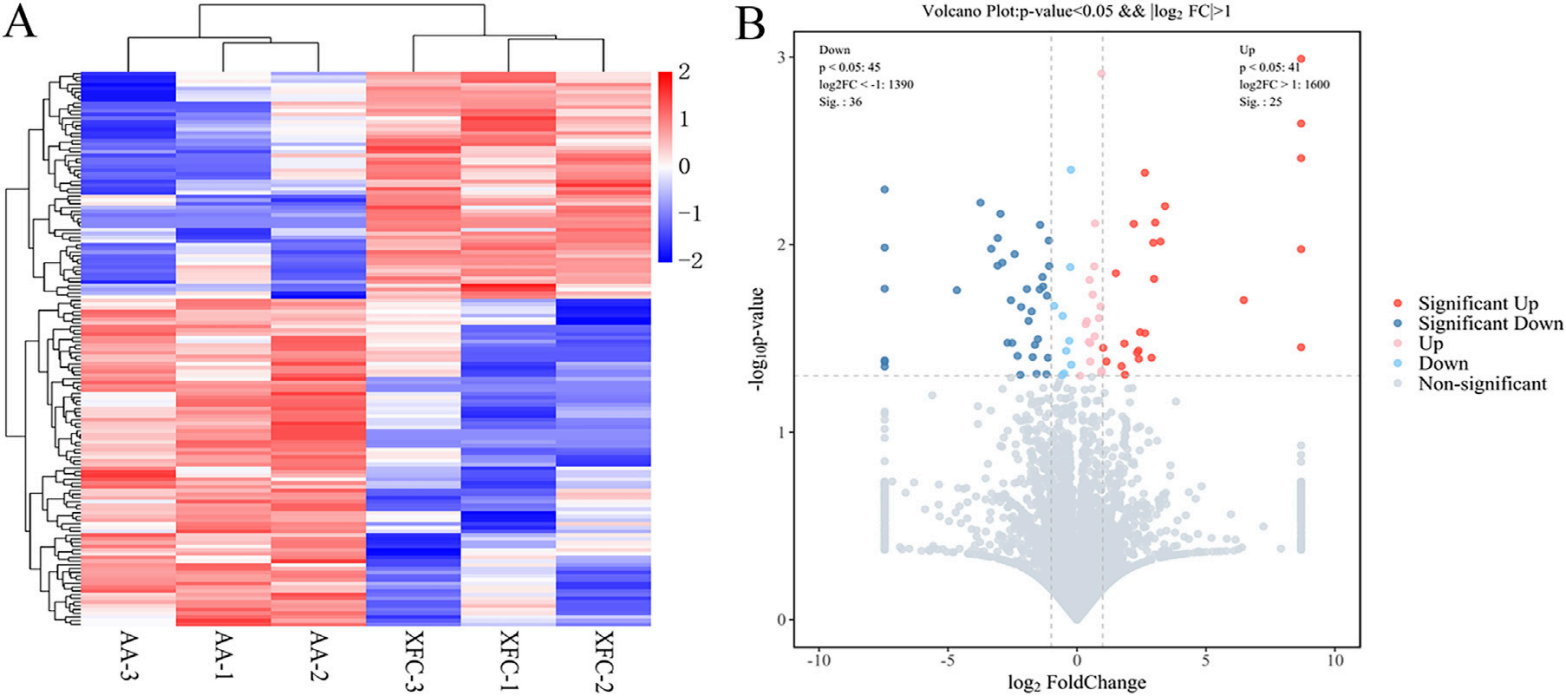
Proteomics analysis of lung tissues from rats in the XFC and AA groups. **(A)** The heat map of 86 differentially expressed proteins (DEPs) between the XFC group and the AA group. Upregulation was represented in red and downregulation was represented in blue. **(B)** The volcano scatter plot of 86 DEPs between the XFC group and the AA group. Upregulation was represented in red, downregulation was represented in blue.

Using a threshold of |log2FC| >1 and P < 0.05, we identified 61 DEPs in XFC-treated rats compared with the AA group. Of these, 25 proteins were upregulated and 36 were downregulated. The top five upregulated DEPs were Tpm3, Cnn1, Hdhd3, Phrf1, and Ist1, while the top five downregulated DEPs were Nup43, HMGCS2, Aaas, Fhip2a, and Cul2 (Table 2). These changes were visualized in a heatmap and a volcano scatter plot (Figures 4A,B).

**TABLE 2:** The top five upregulated and downregulated DEPs.

ID	Symbol	log_2_FC	p.value	Up/Down
NP_001017454.1	Ist1	2.959316936	0.00977505	Up
NP_620793.1	Phrf1	3.032184831	0.007635059	Up
NP_001102981.1	Hdhd3	3.237579051	0.009636475	Up
NP_113935.1	Cnn1	3.414117838	0.006237351	Up
NP_775134.1	Tpm3	6.465309243	0.019766089	Up
XP_006233064.1	Hmgcs2	−4.656967805	0.017512113	Down
NP_001121663.1	Nup43	−3.741195401	0.00597892	Down
XP_006242449.1	Aaas	−3.322901373	0.010534205	Down
XP_342069.4	Fhip2a	−3.076531694	0.012954256	Down
NP_001101887.1	Cul2	−2.894494852	0.01248761	Down

### 3.5 Protein function classification and enrichment analysis

To elucidate the biological functions and pathways associated with the 61 identified DEPs, comprehensive GO and KEGG analyses were performed. Additionally, to better understand the interactions between DEPs, the STRING database was queried to identify PPIs.

**FIGURE 5:**
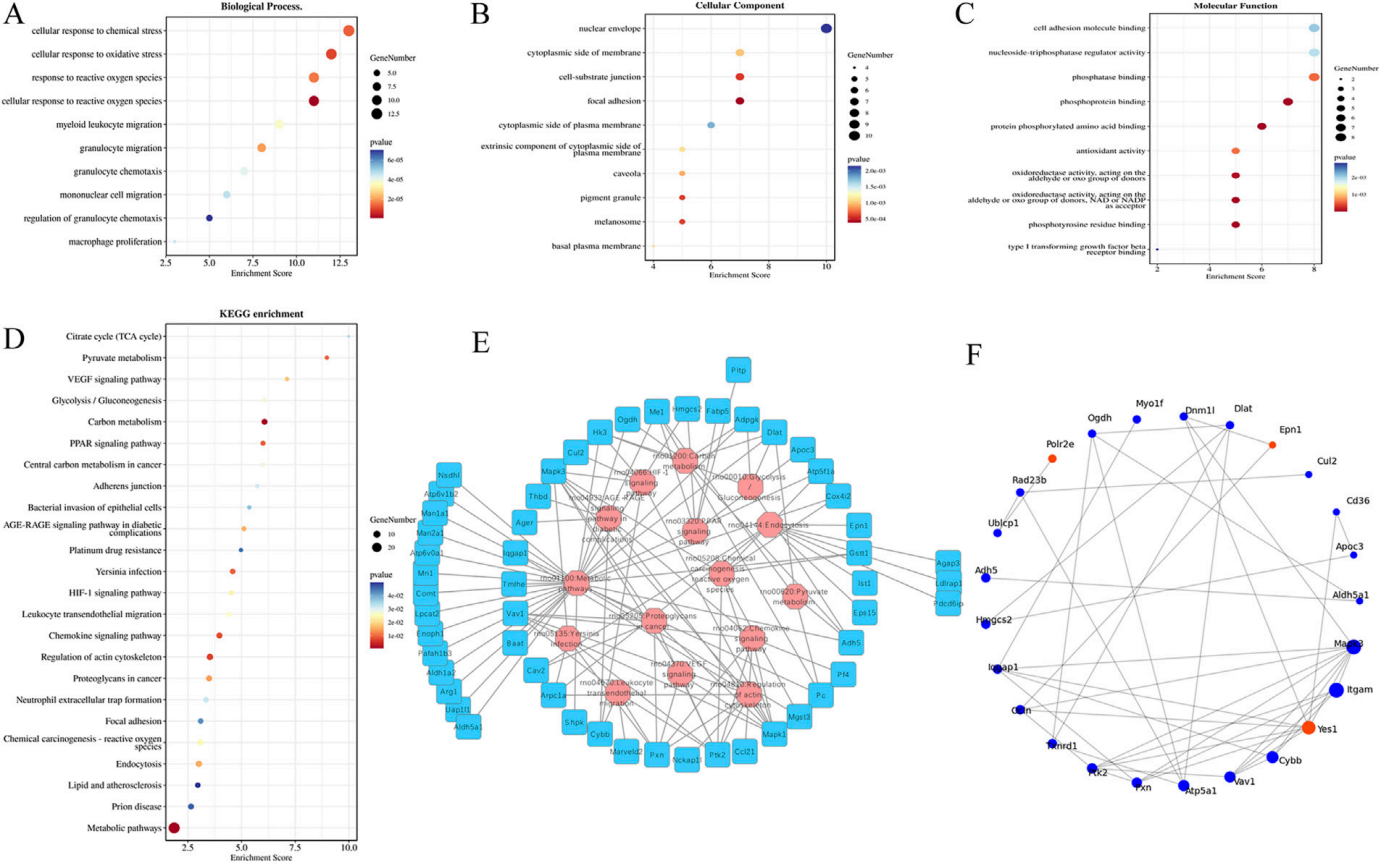
Protein function classification and enrichment analysis between the XFC group and the AA group. **(A)** GO analysis of the biological process. **(B)** GO analysis of the cellular component. **(C)** GO analysis of molecular function. **(D,E)** KEGG pathway annotation and enrichment. **(F)** PPI analysis of the top 25 DEPs.

GO analysis was conducted in three main categories: BP, CC, and MF. In BP (Figure 5A), significant changes were observed in cellular response to ROS, cellular response to oxidative stress, cellular response to chemical stress, granulocyte migration, myeloid leukocyte migration, granulocyte chemotaxis, macrophage proliferation, mononuclear cell migration, and regulation of granulocyte chemotaxis. In CC (Figure 5B), notable changes were detected in focal adhesion, cell–substrate junction, melanosome, pigment granule, caveola, cytoplasmic side of the membrane, basal plasma membrane, extrinsic component of the cytoplasmic side of the plasma membrane, and nuclear envelope. In MF (Figure 5C), the most affected functional categories included protein phosphorylated amino acid binding, phosphoprotein binding, phosphotyrosine residue binding, oxidoreductase activity acting on aldehyde or oxo groups of donors with NAD or NADP as acceptors, phosphatase binding, antioxidant activity, nucleoside-triphosphatase regulator activity, cell adhesion molecule binding, and type I transforming growth factor beta receptor binding. These results suggest that XFC may alleviate lung injury in AA rats primarily by regulating cell adhesion processes and reducing oxidative stress.

KEGG pathway analysis further revealed the main pathways associated with DEPs in the XFC group. The most significantly altered pathways included metabolic pathways, the PPAR signaling pathway, the HIF-1 signaling pathway, chemical carcinogenesis–ROS, pyruvate metabolism, glycolysis/gluconeogenesis, and focal adhesion. Among these, the PPAR signaling pathway, which is closely linked to oxidative stress, was enriched with five proteins: HMGCS2, Me1, Fabp5, Pltp, and Apoc3. Of these, HMGCS2 was the most downregulated DEP (Figures 5D,E).

Furthermore, PPI analysis of DEPs was conducted using Cytoscape software to identify potential XFC targets in the treatment of lung injury in AA rats. The resulting network comprised 25 proteins, which were considered key proteins for further investigation. Notably, HMGCS2 and Apoc3, both associated with oxidative stress responses, were significantly downregulated and enriched in the PPAR signaling pathway (Figure 5F). These findings suggest that XFC may attenuate lung injury in AA rats by modulating the PPAR signaling pathway to counteract oxidative stress.

### 3.6 XFC activated the PPARγ signaling pathway and inhibited oxidative stress in the lung tissue of AA rats

**FIGURE 6:**
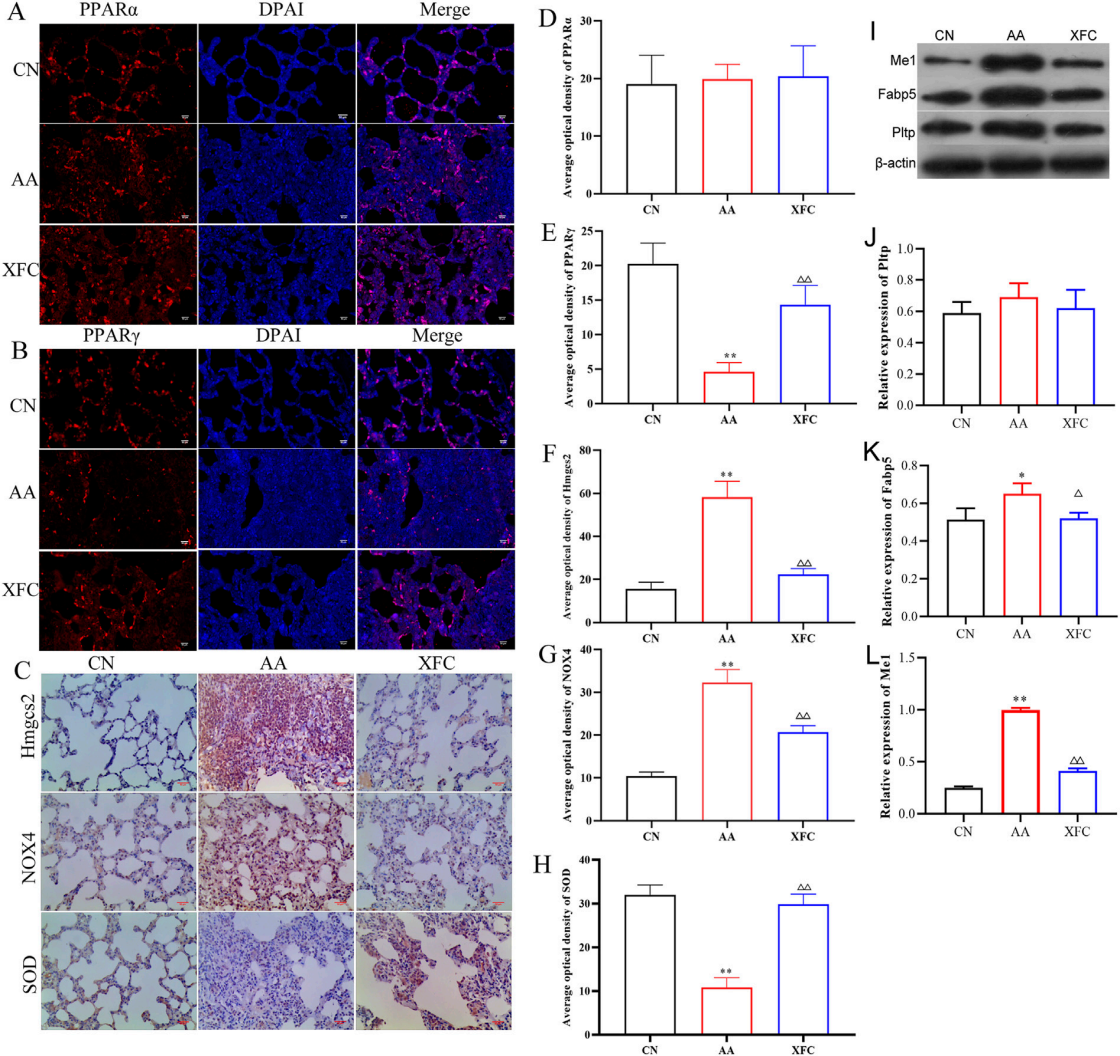
XFC activated PPARγ signaling pathway and inhibited oxidative stress response. **(A,D)** Immunofluorescence representative image and average optical density of Peroxisome Proliferator-Activated Receptor Alpha (PPARα) (n = 3). **(B,E)** Immunofluorescence representative image and average optical density of PPARγ (n = 3). **(C,F,G,H)** Immunohistochemical representative image and average optical density of 3- hydroxy-3-methylglutaryl-Coenzyme A synthase 2 (mitochondrial) (Hmgcs2), NADPH Oxidase 4(NOX4), and Superoxide Dismutase(SOD) (n = 3). **(I–L)** Western blot representative image and relative expression levels of Malic Enzyme 1(Me1), Fatty Acid-Binding Protein 5(Fabp5) and Phospholipid Transfer Protein(Pltp) (n = 3). *P < 0.05 or **P < 0.01 vs. CN group, ^△^ *P* < 0.05 or ^△△^ *P* < 0.01 vs. AA group. CN group: SD rats. AA group: Rats successfully modeled by the Fauquier complete adjuvant method. XFC group: Rats with AA treated by high-dose XFC intragastric administration.

To determine whether XFC alleviates lung injury by reducing oxidative stress through the PPAR signaling pathway, we examined changes in PPAR-related proteins and oxidative stress markers following XFC intervention. Compared with the AA group, the expression of PPARγ protein in the XFC group was significantly increased, whereas no significant difference was observed in PPARα levels (Figures 6A,B,D,E). These results indicate that XFC likely activates the PPAR signaling pathway through PPARγ rather than PPARα. Additionally, the protein levels of HMGCS2, Me1, Fabp5, NOX4, and SOD were significantly decreased in the XFC group (Figures 6C–L), suggesting that XFC reduces the accumulation of oxidants in lung tissue while enhancing the antioxidant capacity of the lungs.

### 3.7 XFC alleviates inflammation and oxidative stress to prevent fibrosis via the PPARγ signaling pathway in HFL-1 cells

**FIGURE 7:**
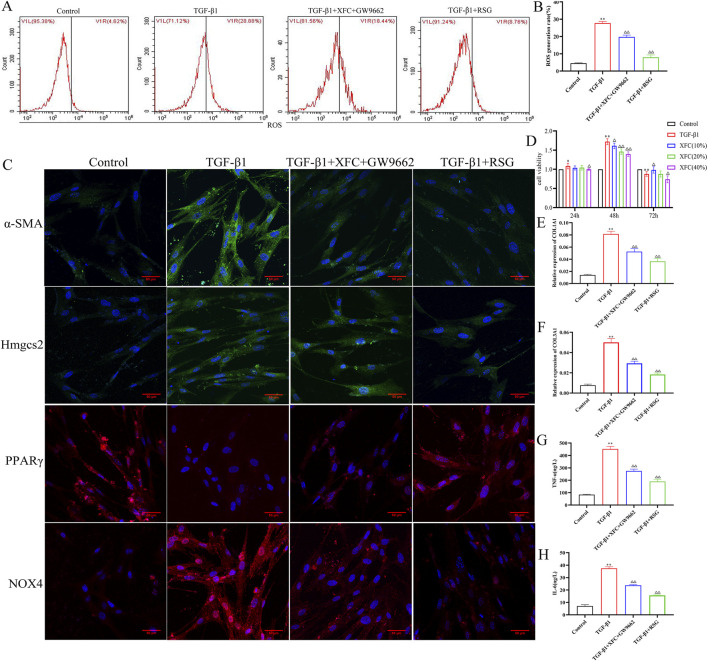
XFC alleviates inflammation and oxidative stress to anti-fibrosis through PPARγ signaling pathway in HFL-1 cells. **(A,B)** Flow cytometry representative images and generation rate of ROS(n = 3). **(C)** Immunofluorescence representative images of Hmgcs2, PPARγ, NOX4, and α-SMA(n = 3). **(D)** Cell viability of HFL-1 cells (n = 6). **(E–H)** Relative expression of COL1A1, COL3A1, TNF-α, and IL-6 (n = 3). *P < 0.05 or **P < 0.01 vs. Control group, ^△^ *P* < 0.05 or ^△△^ *P* < 0.01vs TGF-β1 group. Control group: HFL-1 cells. TGF-β1 group: HFL-1 cells treat with TGF-β1 for 48h. TGF-β1+XFC + GW9662 group: HFL-1 cells treat with TGF-β1, XFC and GW9662 for 48h. TGF-β1+RSG group: HFL-1 cells treat with TGF-β1 and RSG for 48h.

To further confirm that XFC reduces inflammation and oxidative stress to prevent pulmonary fibrosis by regulating the PPARγ signaling pathway, an *in vitro* cell model was established using TGF-β1-induced HFL-1 cells. After TGF-β1 stimulation, PPARγ protein levels were significantly reduced, while HMGCS2, ROS, NOX4, α-SMA, COL1A1, COL3A1, TNF-α, and IL-6 levels were markedly increased, confirming the successful establishment of the model. Subsequent treatment of TGF-β1-induced HFL-1 cells with RSG, a PPARγ agonist, 10 μmol/L for 48 h, upregulated PPARγ protein expression while decreasing the levels of HMGCS2, ROS, NOX4, α-SMA, COL1A1, COL3A1, TNF-α, and IL-6. In addition, co-treatment with GW9662 (a PPARγ antagonist at 5 μmol/L) and XFC-containing serum for 48 h demonstrated that XFC-containing serum could counteract the pro-inflammatory and oxidative effects of GW9662. Specifically, this treatment restored PPARγ protein expression and reduced the expression of HMGCS2, ROS, NOX4, α-SMA, COL1A1, COL3A1, TNF-α, and IL-6. In conclusion, the XFC-containing serum has similar functions to RSG (Figures 7A-H).

## 4 Discussion

RA is a chronic systemic autoimmune disorder characterized by persistent inflammation. Although primarily associated with joint destruction, RA is frequently accompanied by extra-articular manifestations, with pulmonary complications being particularly common and severe. Epidemiological studies indicate that approximately 70% of RA patients exhibit some form of lung involvement, with ILD representing one of the most prevalent and serious complications, occurring in up to 47% of cases (Olson et al., 2011; Raimundo et al., 2019). Early-stage RA-ILD may present as alveolitis, which can often be detected through abnormal chest radiographs and pulmonary function tests. In advanced stages, the disease can progress to interstitial fibrosis, a major contributor to poor prognosis and increased mortality (Chang et al., 2023). Early diagnosis and timely intervention are therefore critical, yet they are often delayed or overlooked. Current pharmacological management of RA-ILD includes glucocorticoids, immunosuppressive agents, cytotoxic drugs, anti-fibrotic therapies, and novel biologics (Cassone et al., 2020). However, long-term use of glucocorticoids and immunosuppressants is associated with significant adverse effects, including gastrointestinal, hepatic, and renal toxicity, as well as the development of drug resistance (Lo et al., 2022). In addition, anti-fibrotic and targeted therapies are costly, demonstrate variable efficacy, and impose a considerable economic burden (Baker et al., 2023). These limitations highlight an unmet clinical need for safer, more effective, and affordable treatment options.

In recent years, TCM has attracted increasing attention as a potential therapeutic approach for RA-ILD (Yang et al., 2024). Unlike single-target synthetic drugs, TCM formulations typically consist of multiple active compounds that act through diverse mechanisms, offering potential advantages in treating multifactorial diseases such as RA-ILD.

According to Professor Liu of Anhui University of TCM, RA-related lung injury is attributed in TCM theory to spleen deficiency, which leads to the accumulation of internal phlegm and stasis in the lungs, manifesting as inflammation and fibrosis. Based on this principle, the formula XFC was developed. XFC is a multi-herb TCM prescription that has been extensively studied over the past two decades. Both clinical and basic research provide evidence supporting its efficacy in ameliorating RA-induced lung injury. A randomized controlled trial demonstrated that XFC not only alleviated joint symptoms but also significantly improved pulmonary ventilation and diffusion capacity in patients with RA (14). Previous animal studies further indicated that XFC improves lung function in adjuvant-induced arthritis (AA) rats, potentially through modulation of regulatory T-cell balance and the Notch signaling pathway (Wan et al., 2012; Wan et al., 2014). However, its effects on interstitial fibrosis and the underlying mechanisms remain unclear.

In this study, we employed LC-MS-based untargeted metabolomics and TMT-based proteomic sequencing to investigate the therapeutic mechanisms of XFC in an AA rat model of RA-associated lung injury. Phytochemical analysis revealed that the alcohol extract of XFC contains flavonoids, flavones, isoflavones, fatty acids, alkaloids, benzodiazepines, and other compounds. Notable high-abundance constituents include quercetin (Sang et al., 2022; Sul and Ra, 2021), caffeic acid (Huang et al., 2024), astragalosides (Yu J. Z. et al., 2022; Zhang M. et al., 2023), kaempferol (Yang et al., 2021; Yang et al., 2020), formononetin (Aladaileh et al., 2019; Ouyang et al., 2023), betaine (Jorgačević et al., 2022), epicatechin (Shariati et al., 2019), ononin (Dong et al., 2022), luteolin (F. Li et al., 2022), berberine (Z. Yang et al., 2023), and celastrol (Zhou et al., 2022)—many of which are known for their anti-inflammatory, antioxidant, anti-fibrotic, and lung-protective properties. Among these, astragalosides (Jiang et al., 2021) and celastrol (Yang et al., 2022) are particularly noteworthy, having been confirmed to exert anti-inflammatory and immunomodulatory effects in the AA model. These findings suggest that the pharmacological activity of XFC is mediated by multiple compounds rather than relying on a single compound.

To evaluate the effects of XFC on RA-associated lung injury, an AA rat model (Song et al., 2016) was established using FCA, and high- and low-dose XFC treatments were administered. LEF was used as a positive control due to its documented efficacy in reducing oxidative stress, alveolar inflammation, and fibrosis (Kayhan et al., 2013). Our results showed that FCA-induced AA rats developed significant lung injury after 30 days, characterized by impaired lung function, disrupted alveolar architecture, inflammatory cell infiltration, and elevated levels of inflammatory cytokines and fibrotic markers (COL1A1, COL13A, and α-SMA). XFC treatment significantly reduced paw swelling, arthritis scores, and histopathological scores, with the high dose producing the most pronounced effect. Furthermore, XFC markedly improved lung function, alleviated alveolar damage and inflammatory infiltration, decreased cytokine levels in bronchoalveolar lavage fluid, and downregulated fibrotic markers. These results suggest that XFC mitigates lung injury in AA rats, likely by inhibiting inflammation and fibrosis.

Proteomic analysis of lung tissues identified 61 DEPs between the XFC-treated and model groups. Among these, HMGCS2 was significantly downregulated. GO and KEGG enrichment analyses indicated that the most affected BP and pathways included cellular response to ROS, cellular response to oxidative stress, and the PPAR signaling pathway. PPI network analysis further identified HMGCS2 and Apoc3 as hub proteins, suggesting their central role in the therapeutic mechanism of XFC.

Oxidative stress is a key driver of inflammatory responses and acute/chronic lung injury (Bezerra et al., 2023; Dhlamini et al., 2022). Following pulmonary damage, recruited inflammatory cells and structural cells (e.g., epithelial cells and fibroblasts) generate endogenous ROS, thereby contributing to tissue destruction (Kellner et al., 2017). Mitochondrial respiration and NOX4 are major sources of ROS (Fan et al., 2023; Mazat et al., 2020). NOX4 expression is elevated in pulmonary fibroblasts during fibrosis, and its knockdown ameliorates bleomycin-induced fibrosis in mice (Yu L. et al., 2022). SOD is a critical antioxidant enzyme that mitigates oxidative damage (Liu et al., 2023; Rosa et al., 2021).

Peroxisome proliferator-activated receptors are ligand-activated nuclear hormone receptors that regulate diverse physiological processes (Mirza et al., 2019). Three subtypes exist: PPARα, PPARγ, and PPARβ/δ. PPARα is expressed in the bronchial epithelium, alveolar walls, and macrophages. In contrast, PPARγ is widely expressed in lung fibroblasts, airway epithelial cells, type II pneumocytes, macrophages, T cells, and smooth muscle cells (Braissant et al., 1996; Simon et al., 2006). PPARβ/δ has not been detected in lung cells. PPARγ can be activated by synthetic ligands such as RSG or pioglitazone, which exert anti-inflammatory, anti-fibrotic, and cytoprotective effects. PPARγ activation inhibits TGF-β–induced fibrotic responses (Xiang et al., 2023) and suppresses the release of pro-inflammatory cytokines (e.g., IL-6, IL-1β, and TNF-α) in airway epithelial cells (Pan et al., 2021). In bleomycin-induced pulmonary fibrosis models, PPARγ agonists have been shown to attenuate weight loss, improve histopathological outcomes, and enhance survival (Kabaliei et al., 2024). They also regulate fibroblast-to-myofibroblast differentiation(Kulkarni et al., 2011) and reduce airway remodeling in asthma models (Zhao et al., 2014). Moreover, PPARγ promotes antioxidant defense by enhancing SOD expression through PPAR response elements (Dovinova et al., 2020).

HMGCS2 catalyzes the condensation of acetyl-CoA and acetoacetyl-CoA to form HMG-CoA, a key irreversible step in ketogenesis. Multiple transcription factors regulate its expression. PPARγ has been shown to regulate HMGCS2 in hyperlipidemia-induced cardiomyocytes and colon cancer cells (Kim et al., 2019; Sikder et al., 2018). HMGCS2 promotes cell invasion and metastasis by enhancing PPARα-mediated transcriptional activity of *Src* (Chen et al., 2017). Additionally, PPARα/HMGCS2 signaling may induce ferroptosis (Duan et al., 2021). Wang et al. demonstrated that HMGCS2 overexpression promotes ROS accumulation and mitochondrial membrane potential loss, thereby inducing diabetic cardiomyopathy (Wang et al., 2020). Conversely, Chen et al. reported that silencing HMGCS2 alleviates high glucose-induced diabetic cardiomyopathy *in vitro* by enhancing cell viability, inhibiting apoptosis, suppressing inflammatory response, and reducing oxidative stress (69). Furthermore, PPARγ acts as an upstream regulator of HMGCS2: PPARγ activation suppresses HMGCS2 expression, whereas PPARγ inhibition elevates it (70).

Our findings demonstrate that XFC activates PPARγ signaling, resulting in the downregulation of HMGCS2, a reduction in oxidative stress, and the attenuation of inflammatory and fibrotic responses. This mechanism is consistent with studies on synthetic PPARγ agonists such as RSG, which have shown efficacy in preclinical models of lung fibrosis (Kabaliei et al., 2024; Kulkarni et al., 2011; Xiang et al., 2023). However, clinical application of synthetic PPARγ agonists is limited by systemic side effects, including weight gain, fluid retention, and cardiovascular risks (Nissen and Wolski, 2007). By contrast, XFC, with its multi-component composition, may offer a more favorable efficacy–safety profile. Although both XFC and RSG share PPARγ activation as a common mechanism, LC-MS analysis indicates that XFC provides a combination of bioactive compounds (e.g., astragaloside IV, quercetin, kaempferol, and triptolide) with complementary anti-inflammatory, antioxidant, and immunomodulatory properties (Jiang et al., 2021; Yang et al., 2024; Zhang M. et al., 2023; Zhou et al., 2022). Rather than directly and potently activating PPARγ, XFC appears to modulate the pathway in a more balanced and tissue-specific manner, while simultaneously engaging additional anti-fibrotic and anti-inflammatory pathways. This multi-target strategy is characteristic of TCM and may provide a more comprehensive and sustainable therapeutic approach for complex conditions such as RA-associated lung injury.

Notably, the TCM theory proposed by Professor Liu—that RA-associated lung injury arises from spleen deficiency leading to phlegm-stasis accumulation—aligns closely with our molecular findings. In TCM, the spleen governs transformation and transport; its dysfunction implies metabolic dysregulation. XFC’s reduction of oxidative stress (via decreased NOX4 and ROS) and enhancement of antioxidant capacity (via increased SOD) can be interpreted as rectifying spleen deficiency and eliminating pathogenic phlegm-stasis. The modern correlates of “phlegm” and “stasis”—namely, inflammatory infiltration, cytokine release, and collagen deposition—were consistently reduced by XFC treatment. Thus, PPARγ activation serves as a mechanistic bridge between TCM theory and contemporary pathophysiology. By activating PPARγ and normalizing HMGCS2-mediated metabolic dysfunction, XFC addresses the core TCM syndrome of spleen deficiency and its pathological consequences (inflammation/fibrosis).

In summary, this study confirms that XFC upregulates PPARγ expression, downregulates HMGCS2, and reduces oxidative stress markers (NOX4, ROS) as well as fibrotic indicators in AA rat lungs. Furthermore, using a pulmonary fibroblast model, we demonstrated that XFC inhibits fibrosis through PPARγ-mediated suppression of inflammation and oxidative stress.

Nonetheless, several limitations remain. First, although the AA rat model is widely used in experimental research, it does not fully recapitulate the complex immunological and pathological features of human-associated lung injury, which may impede the direct translation of our findings into clinical applications. Second, while our results—initiated by proteomic analysis—suggest the involvement of the PPARγ/HMGCS2 pathway in regulating oxidative stress and inflammatory responses, future rescue experiments utilizing HMGCS2 knockdown or overexpression will be essential to establish a direct causal relationship between this pathway and the observed outcomes, as well as to clarify its role in the development of lung injury in AA rats. Third, the relatively short duration of this study may have limited our ability to capture long-term fibrotic changes fully. Finally, the pharmacokinetic profile of the individual bioactive components within XFC and the long-term sustainability of its therapeutic effects remain to be elucidated. Therefore, evaluating the long-term effects of XFC on pulmonary fibrosis and remodeling is a key focus of our ongoing and future research. We are currently conducting an extended observational study in animal models and intend to report the long-term impact of XFC on pulmonary fibrosis associated with RA in forthcoming publications. Despite these limitations, our findings provide a solid foundation for further research and potential clinical application.

## 5 Conclusion

In summary, our study indicates that XFC effectively improves lung injury in AA rats and inhibits inflammatory and oxidative stress responses. Through proteomic analysis, along with *in vitro* and *in vivo* experiments, the molecular mechanism of XFC was further elucidated. We found that XFC can partially reverse the effects of PPARγ antagonists, activate the PPARγ signaling pathway, inhibit oxidative stress and inflammatory responses, and exert effects similar to those of PPARγ agonists. Therefore, XFC mitigates inflammation and oxidative stress by regulating the PPARγ/HMGCS2 pathway, thereby counteracting fibrosis and reducing lung injury. This study not only provides experimental evidence for the potential of XFC in the prevention and treatment of RA-associated lung injury but also highlights new molecular targets and pathways for addressing RA-associated interstitial lung fibrosis.

## Data Availability

The datasets presented in this study can be found in online repositories. The names of the repository/repositories and accession number(s) can be found below: https://www.iprox.cn/page/ProjectFileList.html?projectId=IPX0006495000&url=1686278190812oyzK, IPX0006495000.

## Glossary

AA: adjuvant arthritis
Apoc3: apolipoprotein C-III
α-SMA: a-smooth muscle actin
Aaas: achalasia, adrenocortical insufficiency, alacrimia (Allgrove, triple-A)
BP: biological processes
BLM: bleomycin
CC: cellular components
COL1A1: collagen type 1A1
COL3A1: collagen type 3A1
Cnn1: calponin 1
Cul2: cullin 2
DEPs: differentially expressed proteins
FVC: forced vital capacity
FEV0.2: forced expiratory volume at 0.2 s
FEV0.2/FVC: forced expiratory volume at 0.2 s/forced vital capacity
FEF50: expiratory flow 50%
Fhip2a: FHF complex subunit Hook Interacting Protein 2a
Fabp5: fatty acid-binding protein 5
GO: gene ontology
Hdhd3: haloacid dehalogenase-like hydrolase domain containing 3
Hmgcs2: mitochondrial 3-hydroxy-3-methylglutaryl CoA synthase 2
KEGG: kyoto encyclopedia of genes and genomes
LC-MS: liquid chromatography-mass spectrometry
ILD: interstitial lung disease
Ist1: increased sodium tolerance-1
MF: molecular functions
Me1: Malic Enzyme 1
Nup43: nucleoporin 43
NOX4: nicotinamide adenine dinucleotide phosphate oxidase 4
PEF: peak expiratory flow
Phrf1: human PHD and RING finger domain-containing protein 1
Pltp: recombinant phospholipid transfer protein
PPI: protein-protein interaction
PPARα: peroxisome proliferators activated receptor α
PPARγ: peroxisome proliferators activated receptor γ
RA: rheumatoid arthritis
RSG: rosiglitazone
ROS: reactive oxygen
SOD: superoxide dismutase
TMT: tandem mass tags
TCM: traditional Chinese medicine
Tpm3: tropomyosin 3
XFC: Xinfeng Capsule

## References

[B1] Akiyama M. Kaneko Y. (2022). Pathogenesis, clinical features, and treatment strategy for rheumatoid arthritis-associated interstitial lung disease. Autoimmun. Rev. 21 (5), 103056. 10.1016/j.autrev.2022.103056 35121155

[B2] Aladaileh S. Hussein O. Abukhalil M. Saghir S. Bin-Jumah M. Alfwuaires M. (2019). Formononetin upregulates Nrf2/HO-1 signaling and prevents oxidative stress, inflammation, and kidney injury in methotrexate-induced rats. Antioxidants (Basel) 8 (10), 430. 10.3390/antiox8100430 31561418 PMC6827027

[B3] Baker M. C. Liu Y. Lu R. Lin J. Melehani J. Robinson W. H. (2023). Incidence of interstitial lung disease in patients with rheumatoid arthritis treated with biologic and targeted synthetic disease-modifying antirheumatic drugs. JAMA Netw. Open 6 (3), e233640. 10.1001/jamanetworkopen.2023.3640 36939701 PMC10028485

[B4] Bezerra F. S. Lanzetti M. Nesi R. T. Nagato A. C. Silva C. P. e. Kennedy-Feitosa E. (2023). Oxidative stress and inflammation in acute and chronic lung injuries. Antioxidants 12 (3), 548. 10.3390/antiox12030548 36978796 PMC10045332

[B5] Braissant O. Foufelle F. Scotto C. Dauça M. Wahli W. (1996). Differential expression of peroxisome proliferator-activated receptors (PPARs): tissue distribution of PPAR-alpha,-beta, and-gamma in the adult rat. Endocrinology 137 (1), 354–366. 10.1210/endo.137.1.8536636 8536636

[B6] Cassone G. Manfredi A. Vacchi C. Luppi F. Coppi F. Salvarani C. (2020). Treatment of rheumatoid arthritis-associated interstitial lung disease: lights and shadows. J. Clin. Med. 9 (4), 1082. 10.3390/jcm9041082 32290218 PMC7230307

[B7] Chang S. H. Lee J. S. Ha Y.-J. Kim M. U. Park C. H. Lee J. S. (2023). Lung function trajectory of rheumatoid arthritis–associated interstitial lung disease. Rheumatology 62 (9), 3014–3024. 10.1093/rheumatology/kead027 36702465 PMC10473227

[B8] Chen S.-W. Chou C.-T. Chang C.-C. Li Y.-J. Chen S.-T. Lin I.-C. (2017). HMGCS2 enhances invasion and metastasis *via* direct interaction with PPARα to activate Src signaling in colorectal cancer and oral cancer. Oncotarget 8 (14), 22460–22476. 10.18632/oncotarget.13006 27816970 PMC5410236

[B9] Dai Y. Wang W. Yu Y. Hu S. (2021). Rheumatoid arthritis–associated interstitial lung disease: an overview of epidemiology, pathogenesis and management. Clin. Rheumatol. 40, 1211–1220. 10.1007/s10067-020-05320-z 32794076

[B10] Dhlamini Q. Wang W. Feng G. Chen A. Chong L. Li X. (2022). FGF1 alleviates LPS-induced acute lung injury *via* suppression of inflammation and oxidative stress. Mol. Med. 28 (1), 73. 10.1186/s10020-022-00502-8 35764933 PMC9238076

[B11] Dong L. Yu L. Liu A. Alahmadi T. A. Almoallim H. S. Durairaj K. (2022). Ononin mitigates streptozotocin-induced diabetic nephropathy in rats *via* alleviating oxidative stress and inflammatory markers. J. King Saud University-Science 34 (6), 102029. 10.1016/j.jksus.2022.102029

[B12] Dovinova I. Kvandova M. Balis P. Gresova L. Majzunova M. Horakova L. (2020). The role of Nrf2 and PPARγ in the improvement of oxidative stress in hypertension and cardiovascular diseases. Physiological Res. 69 (Suppl. 4), S541–S553. 10.33549/physiolres.934612 33656904 PMC8603703

[B13] Duan J.-Y. Lin X. Xu F. Shan S.-K. Guo B. Li F.-X.-Z. (2021). Ferroptosis and its potential role in metabolic diseases: a curse or revitalization? Front. Cell Dev. Biol. 9, 701788. 10.3389/fcell.2021.701788 34307381 PMC8299754

[B14] Fan X. Dong T. Yan K. Ci X. Peng L. (2023). PM2. 5 increases susceptibility to acute exacerbation of COPD *via* NOX4/Nrf2 redox imbalance-mediated mitophagy. Redox Biol. 59, 102587. 10.1016/j.redox.2022.102587 36608590 PMC9813701

[B15] Finckh A. Gilbert B. Hodkinson B. Bae S.-C. Thomas R. Deane K. D. (2022). Global epidemiology of rheumatoid arthritis. Nat. Rev. Rheumatol. 18 (10), 591–602. 10.1038/s41584-022-00827-y 36068354

[B16] Gao L. Wang F. Meng M. (2020). Chromatographic fingerprinting and quantitative analysis for the quality evaluation of xinfeng capsule. Acta Chromatogr. 33 (1), 37–43. 10.1556/1326.2020.00743

[B17] Hazra S. Peebles K. A. Sharma S. Mao J. T. Dubinett S. M. (2008). The role of PPARgamma in the cyclooxygenase pathway in lung cancer. PPAR Res. 2008 (1), 790568. 10.1155/2008/790568 18769553 PMC2526169

[B18] Huang S. Kronzer V. L. Dellaripa P. F. Deane K. D. Bolster M. B. Nagaraja V. (2020). Rheumatoid arthritis–associated interstitial lung disease: current update on prevalence, risk factors, and pharmacologic treatment. Curr. Treat. options rheumatology 6, 337–353. 10.1007/s40674-020-00160-z 33282632 PMC7709915

[B19] Huang C.-W. Lee S.-Y. Du C.-X. Wu S.-T. Kuo Y.-H. Ku H.-C. (2024). Caffeic acid ethanolamide induces antifibrosis, anti-inflammatory, and antioxidant effects protects against bleomycin-induced pulmonary fibrosis. Biomed. and Pharmacother. 173, 116298. 10.1016/j.biopha.2024.116298 38394850

[B20] Jian L. Yuan W. Chuanbing H. Jianhua X. Zhijun L. Liang X. (2015). Efficacy and safety of xinfeng capsule in patients with rheumatoid arthritis: a multi-center parallel-group double-blind randomized controlled trial. J. Traditional Chin. Med. 35 (5), 487–498. 10.1016/s0254-6272(15)30130-8 26591677

[B21] Jiang H. Fan C. Lu Y. Cui X. Liu J. (2021). Astragaloside regulates lncRNA LOC100912373 and the miR-17-5p/PDK1 axis to inhibit the proliferation of fibroblast-like synoviocytes in rats with rheumatoid arthritis. Int. J. Mol. Med. 48 (1), 130. 10.3892/ijmm.2021.4963 34013364 PMC8136124

[B22] Jorgačević B. Stanković S. Filipović J. Samardžić J. Vučević D. Radosavljević T. (2022). Betaine modulating MIF-Mediated oxidative stress, inflammation and fibrogenesis in thioacetamide-induced nephrotoxicity. Curr. Med. Chem. 29 (31), 5254–5267. 10.2174/0929867329666220408102856 35400322

[B23] Kabaliei A. Izmailova O. Palchyk V. Shinkevich V. Shlykova O. (2024). The pparg receptor agonist pioglitazone reduces pulmonary fibrosis induced by bleomycin administration in mice. Med. Ecol. Problems 28 (3), 19–27. 10.31718/mep.2024.28.3.03

[B24] Kayhan S. Guzel A. Duran L. Tutuncu S. Guzel A. Gunaydın M. (2013). Effects of leflunomide on inflamation and fibrosis in bleomycine induced pulmonary fibrosis in wistar albino rats. J. Thorac. Dis. 5 (5), 641–649. 10.3978/j.issn.2072-1439.2013.09.20 24255778 PMC3815717

[B25] Kellner M. Noonepalle S. Lu Q. Srivastava A. Zemskov E. Black S. M. (2017). “ROS signaling in the pathogenesis of acute lung injury (ALI) and acute respiratory distress syndrome (ARDS),” in Pulmonary vasculature redox signaling in health and disease, 105–137. 10.1007/978-3-319-63245-2_8 PMC7120947 29047084

[B26] Kim J. T. Li C. Weiss H. L. Zhou Y. Liu C. Wang Q. (2019). Regulation of ketogenic enzyme HMGCS2 by Wnt/β-catenin/PPARγ pathway in intestinal cells. Cells 8 (9), 1106. 10.3390/cells8091106 31546785 PMC6770209

[B27] Kulkarni A. A. Thatcher T. H. Olsen K. C. Maggirwar S. B. Phipps R. P. Sime P. J. (2011). PPAR-γ ligands repress TGFβ-induced myofibroblast differentiation by targeting the PI3K/Akt pathway: implications for therapy of fibrosis. PloS one 6 (1), e15909. 10.1371/journal.pone.0015909 21253589 PMC3017065

[B28] Li F. Wei R. Huang M. Chen J. Li P. Ma Y. (2022). Luteolin can ameliorate renal interstitial fibrosis-induced renal anaemia through the SIRT1/FOXO3 pathway. Food and Funct. 13 (22), 11896–11914. 10.1039/d2fo02477b 36321482

[B29] Li Q. Wei Y. Wei Y. He K. Liao G. Cheng L. (2024). Erythromycin regulates peroxisome proliferator-activated receptor γ to ameliorate cigarette smoke-induced oxidative stress in macrophages. J. Thorac. Dis. 16 (5), 3051–3060. 10.21037/jtd-23-1647 38883674 PMC11170435

[B30] Liu J. Hu S. Zhu B. Shao S. Yuan L. (2020). Grape seed procyanidin suppresses inflammation in cigarette smoke-exposed pulmonary arterial hypertension rats by the PPAR-γ/COX-2 pathway. Nutr. Metabolism Cardiovasc. Dis. 30 (2), 347–354. 10.1016/j.numecd.2019.09.022 31791634

[B31] Liu C. Fan W. Cheng W. X. Gu Y. Chen Y. Zhou W. (2023). Red emissive carbon dot superoxide dismutase nanozyme for bioimaging and ameliorating acute lung injury. Adv. Funct. Mater. 33 (19), 2213856. 10.1002/adfm.202213856

[B32] Lo C.-Y. Wang C.-H. Wang C.-W. Chen C.-J. Huang H.-Y. Chung F.-T. (2022). Increased interleukin-17 and glucocorticoid receptor-β expression in interstitial lung diseases and corticosteroid insensitivity. Front. Immunol. 13, 905727. 10.3389/fimmu.2022.905727 35865549 PMC9294725

[B33] Mazat J.-P. Devin A. Ransac S. (2020). Modelling mitochondrial ROS production by the respiratory chain. Cell. Mol. Life Sci. 77 (3), 455–465. 10.1007/s00018-019-03381-1 31748915 PMC11104992

[B34] Mirza A. Z. Althagafi I. I. Shamshad H. (2019). Role of PPAR receptor in different diseases and their ligands: physiological importance and clinical implications. Eur. J. Med. Chem. 166, 502–513. 10.1016/j.ejmech.2019.01.067 30739829

[B35] Nissen S. E. Wolski K. (2007). Effect of rosiglitazone on the risk of myocardial infarction and death from cardiovascular causes. N. Engl. J. Med. 356 (24), 2457–2471. 10.1056/NEJMoa072761 17517853

[B36] Olson A. L. Swigris J. J. Sprunger D. B. Fischer A. Fernandez-Perez E. R. Solomon J. (2011). Rheumatoid arthritis–interstitial lung disease–associated mortality. Am. J. Respir. Crit. care Med. 183 (3), 372–378. 10.1164/rccm.201004-0622OC 20851924 PMC5450769

[B37] Ouyang B. Deng L. Yang F. Shi H. Wang N. Tang W. (2023). Albumin-based formononetin nanomedicines for lung injury and fibrosis therapy via blocking macrophage pyroptosis. Mater. Today Bio 20, 100643. 10.1016/j.mtbio.2023.100643 37214555 PMC10193015

[B38] Pan K. Lu J. Song Y. (2021). Artesunate ameliorates cigarette smoke-induced airway remodelling via PPAR-γ/TGF-β1/Smad2/3 signalling pathway. Respir. Res. 22, 91–13. 10.1186/s12931-021-01687-y 33757521 PMC7989207

[B39] Raimundo K. Solomon J. J. Olson A. L. Kong A. M. Cole A. L. Fischer A. (2019). Rheumatoid arthritis–interstitial lung disease in the United States: prevalence, incidence, and healthcare costs and mortality. J. rheumatology 46 (4), 360–369. 10.3899/jrheum.171315 30442831

[B40] Rosa A. C. Corsi D. Cavi N. Bruni N. Dosio F. (2021). Superoxide dismutase administration: a review of proposed human uses. Molecules 26 (7), 1844. 10.3390/molecules26071844 33805942 PMC8037464

[B41] Samhouri B. F. Vassallo R. Achenbach S. J. Kronzer V. L. Davis III J. M. Myasoedova E. (2022). Incidence, risk factors, and mortality of clinical and subclinical rheumatoid arthritis–associated interstitial lung disease: a population‐based cohort. Arthritis Care and Res. 74 (12), 2042–2049. 10.1002/acr.24856 34995017 PMC9272096

[B42] Sang A. Wang Y. Wang S. Wang Q. Wang X. Li X. (2022). Quercetin attenuates sepsis-induced acute lung injury via suppressing oxidative stress-mediated ER stress through activation of SIRT1/AMPK pathways. Cell. Signal. 96, 110363. 10.1016/j.cellsig.2022.110363 35644425

[B43] Shariati S. Kalantar H. Pashmforoosh M. Mansouri E. Khodayar M. J. (2019). Epicatechin protective effects on bleomycin-induced pulmonary oxidative stress and fibrosis in mice. Biomed. and Pharmacother. 114, 108776. 10.1016/j.biopha.2019.108776 30903918

[B44] Sikder K. Shukla S. K. Patel N. Singh H. Rafiq K. (2018). High fat diet upregulates fatty acid oxidation and ketogenesis via intervention of PPAR-γ. Cell. Physiology Biochem. 48 (3), 1317–1331. 10.1159/000492091 30048968 PMC6179152

[B45] Simon D. M. Arikan M. C. Srisuma S. Bhattacharya S. Tsai L. W. Ingenito E. P. (2006). Epithelial cell PPAR[gamma] contributes to normal lung maturation. FASEB J. 20 (9), 1507–1509. 10.1096/fj.05-5410fje 16720732

[B46] Song L.-n. Kong X.-d. Wang H.-j. Zhan L.-b. (2016). Establishment of a rat adjuvant arthritis‐interstitial lung disease model. BioMed Res. Int. 2016 (1), 2970783. 10.1155/2016/2970783 26881215 PMC4736313

[B47] Sul O.-J. Ra S. W. (2021). Quercetin prevents LPS-induced oxidative stress and inflammation by modulating NOX2/ROS/NF-kB in lung epithelial cells. Molecules 26 (22), 6949. 10.3390/molecules26226949 34834040 PMC8625571

[B48] Sun Y. Liu J. Wan L. (2016). Effect of Xinfeng capsule on improving pulmonary function in rheumatoid arthritis patients. Zhongguo Zhong xi yi jie he za zhi Zhongguo Zhongxiyi Jiehe Zazhi= Chin. J. Integr. Traditional West. Med. 36 (7), 814–820. 30634208

[B49] Sun Y. Liu J. Xin L. Wen J. Zhou Q. Chen X. (2023). Xinfeng capsule inhibits inflammation and oxidative stress in rheumatoid arthritis by up-regulating LINC00638 and activating Nrf2/HO-1 pathway. J. Ethnopharmacol. 301, 115839. 10.1016/j.jep.2022.115839 36272490

[B50] Wan L. Liu J. Huang C. Wang Y. Shen X. Zhang W. (2012). Effects of xinfeng capsule on pulmonary function based on treg-mediated notch pathway in a rat model of adjuvant arthritis. J. Traditional Chin. Med. 32 (3), 430–436. 10.1016/s0254-6272(13)60050-3 23297568

[B51] Wan L. Liu J. Huang C.-B. Wang Y. Lei L. Liu L. (2013). Effect of tripterygium glycosides on pulmonary function in adjuvant arthritis rats. J. Chin. Med. Assoc. 76 (12), 715–723. 10.1016/j.jcma.2013.08.002 24060529

[B52] Wan L. Liu J. Huang C. Wang Y. Zheng L. (2014). Effect of xinfeng capsule on pulmonary function in a adjuvant arthritis rat model. J. Traditional Chin. Med. 34 (1), 76–85. 10.1016/s0254-6272(14)60058-3 25102695

[B53] Wan L. Liu J. Huang C.-b. Wang Y. Chen X. Zhang W.-d. (2016). Xinfeng Capsule (新风胶囊) for the treatment of rheumatoid arthritis patients with decreased pulmonary function—A randomized controlled clinical trial. Chin. J. Integr. Med. 22, 168–176. 10.1007/s11655-015-2093-6 26818127

[B54] Wan L. Liu J. Huang C. Zhang W. Qi Y. Zhang X. (2017). Xinfeng Capsule improves lung function by regulating Notch/Jagged-HES axis of type II alveolar epithelial cells in adjuvant arthritis rats. Xi bao yu fen zi Mian yi xue za zhi= Chin. J. Cell. Mol. Immunol. 33 (7), 942–946. 28712402

[B55] Wang F. Meng M. Chen L. Wang X. Sun Q. (2014). Development and validation of a high-performance thin-layer chromatographic fingerprint method for the evaluation of QiYi capsules with the reference of myotonin. JPC-Journal Planar Chromatogr. TLC 27 (3), 199–203. 10.1556/jpc.27.2014.3.9

[B56] Wang L. Bi X. Han J. (2020). Silencing of peroxisome proliferator‐activated receptor‐alpha alleviates myocardial injury in diabetic cardiomyopathy by downregulating 3‐hydroxy‐3‐methylglutaryl‐coenzyme A synthase 2 expression. IUBMB life 72 (9), 1997–2009. 10.1002/iub.2337 32734614

[B57] Xiang H. Xiao J. Sun Z. Liu Z. Zhang J. Xiang H. (2023). The anti-fibrotic efficacy of adelmidrol depends on hepatic PPARγ levels. Biomed. and Pharmacother. 165, 115051. 10.1016/j.biopha.2023.115051 37385215

[B58] Yali W. Jian L. Yuan W. Yue S. (2015). Effect of Xinfeng capsule in the treatment of active rheumatoid arthritis: a randomized controlled trial. J. Traditional Chin. Med. 35 (6), 626–631. 10.1016/s0254-6272(15)30150-3 26742305

[B59] Yang C. Yang W. He Z. He H. Yang X. Lu Y. (2020). Kaempferol improves lung ischemia-reperfusion injury via antiinflammation and antioxidative stress regulated by SIRT1/HMGB1/NF-κB axis. Front. Pharmacol. 10, 1635. 10.3389/fphar.2019.01635 32116668 PMC7025570

[B60] Yang C. Yang W. He Z. Guo J. Yang X. Wang R. (2021). Kaempferol alleviates oxidative stress and apoptosis through mitochondria-dependent pathway during lung ischemia-reperfusion injury. Front. Pharmacol. 12, 624402. 10.3389/fphar.2021.624402 33746757 PMC7969663

[B61] Yang J. Liu J. Li J. Jing M. Zhang L. Sun M. (2022). Celastrol inhibits rheumatoid arthritis by inducing autophagy via inhibition of the PI3K/AKT/mTOR signaling pathway. Int. Immunopharmacol. 112, 109241. 10.1016/j.intimp.2022.109241 36116150

[B62] Yang Z. Bian M. Ma J. Dong Y. Yang D. Qiu M. (2023). Berberine regulates pulmonary inflammatory microenvironment and decreases collagen deposition in response to bleomycin‐induced pulmonary fibrosis in mice. Basic and Clin. Pharmacol. and Toxicol. 132 (2), 154–170. 10.1111/bcpt.13818 36433932

[B63] Yang C. Li N. Li X. Zhao L. Xu H. Shi Q. (2024). Efficacy of Zang Bi formula in treating arthritis and its pulmonary complications in Rheumatoid arthritis interstitial lung disease mice. Chin. General Pract. 27 (24), 3015. 10.12114/j.issn.1007-9572.2023.0257

[B64] Yu J.-Z. Wen J. Ying Y. Yin W. Zhang S.-q. Pang W.-L. (2022). Astragaloside trigger autophagy: implication a potential therapeutic strategy for pulmonary fibrosis. Biomed. and Pharmacother. 154, 113603. 10.1016/j.biopha.2022.113603 36942596

[B65] Yu L. Bian X. Zhang C. Wu Z. Huang N. Yang J. (2022). Ginkgolic acid improves bleomycin‐induced pulmonary fibrosis by inhibiting SMAD4 SUMOylation. Oxidative Med. Cell. Longev. 2022 (1), 8002566. 10.1155/2022/8002566 35707278 PMC9192210

[B66] Zhang M. Wang W. Liu K. Jia C. Hou Y. Bai G. (2023). Astragaloside IV protects against lung injury and pulmonary fibrosis in COPD by targeting GTP-GDP domain of RAS and downregulating the RAS/RAF/FoxO signaling pathway. Phytomedicine 120, 155066. 10.1016/j.phymed.2023.155066 37690229

[B67] Zhang P.-H. Wu D.-B. Liu J. Wen J.-T. Chen E.-S. Xiao C.-H. (2023). Proteomics analysis of lung tissue reveals protein makers for the lung injury of adjuvant arthritis rats. Mol. Med. Rep. 28 (3), 163–16. 10.3892/mmr.2023.13051 37449522 PMC10407615

[B68] Zhao Y. Huang Y. He J. Li C. Deng W. Ran X. (2014). Rosiglitazone, a peroxisome proliferator-activated receptor-γ agonist, attenuates airway inflammation by inhibiting the proliferation of effector T cells in a murine model of neutrophilic asthma. Immunol. Lett. 157 (1-2), 9–15. 10.1016/j.imlet.2013.11.004 24269293

[B69] Zhou Y. Li M. Shen T. Yang T. Shi G. Wei Y. (2022). Celastrol targets cullin-associated and neddylation-dissociated 1 to prevent fibroblast–myofibroblast transformation against pulmonary fibrosis. ACS Chem. Biol. 17 (10), 2734–2743. 10.1021/acschembio.2c00099 36076154

