# Correction: Xinfeng Capsule inhibits oxidative stress via regulating the PPARγ /Hmgcs2 signaling pathway in lung tissue of adjuvant arthritis rats

**DOI:** 10.3389/fphar.2026.1746942

**Published:** 2026-01-30

**Authors:** Ping-Heng Zhang, En-Sheng Chen, Jian Liu, Chang-Hong Xiao

**Affiliations:** 1 Department of Nephrology and Rheumatology, The Affiliated TCM Hospital of Guangzhou Medical University, Guangzhou, China; 2 Rheumatology and Immunology Department, Southern Medical University Hospital of Integrated Traditional Chinese and Western Medicine, Southern Medical University, Guangzhou, China; 3 Department of Rheumatology and Immunology, First Affiliated Hospital of Anhui University of Traditional Chinese Medicine, Hefei, China

**Keywords:** Xinfeng Capsule, adjuvant arthritis, lung injury, oxidative stress, PPARγ, Hmgcs2

Affiliation 2 ‘Rheumatology and Immunology Department, Southern Medical University Hospital of Integrated Traditional Chinese and Western Medicine, Southern Medical University, Guangzhou, China’ was erroneously given as ‘Department of Rheumatology, The First Affiliated Hospital of Guangzhou University of Chinese Medicine, Guangzhou, China’.

Authors En-Sheng Chen and Chang-Hong Xiao were erroneously assigned to affiliation 1 ‘Department of Nephrology and Rheumatology, The Affiliated TCM Hospital of Guangzhou Medical University, Guangzhou, China’. This affiliation has now been removed for authors En-Sheng Chen, Chang-Hong Xiao.

Affiliation 2 ‘Rheumatology and Immunology Department, Southern Medical University Hospital of Integrated Traditional Chinese and Western Medicine, Southern Medical University, Guangzhou, China’ was omitted for authors En-Sheng Chen and Chang-Hong Xiao. This affiliation has now been added for authors En-Sheng Chen, Chang-Hong Xiao.

The original article has been updated.

