# Supplementary material for: Xinfeng Capsule inhibits oxidative stress via regulating the PPARγ/ Hmgcs2 signaling pathway in lung tissue of adjuvant arthritis rats
Source: Front Pharmacol. 2025 Oct 8;16:1599745. doi: 10.3389/fphar.2025.1599745 (PMC12541257; doi:10.3389/fphar.2025.1599745)
Supplement: Supplementary file 1 [file Table1.docx]

Table S1. Oligonucleotide sequences for COL1A1 and COL3A1mRNA gene expansion

| Genes | Primer sequence | | Amplicon Size |
| --- | --- | --- | --- |
| β-actin | Forward | 5’-CCCATCTATGAGGGTTACGC-3’ | 150bp |
|  | Reverse | 5’-TTTAATGTCACGCACGATTTC-3’ |  |
| ColⅠα | Forward | 5’-ACCTCAGGGTATTGCTGGAC-3’ | 74bp |
|  | Reverse | 5’-GACCAGGGAAGCCTCTTTCT-3’ |  |
| ColⅢα | Forward | 5’-CCTGCAGGAAAGGATGGAGA-3’ | 92bp |
|  | Reverse | 5’-ATACCAGCTGGGCCTTTGAT-3’ |  |
